# Head movements induced by voluntary neck flexion stabilize sensorimotor synchronization of the finger to syncopated auditory rhythms

**DOI:** 10.3389/fpsyg.2024.1335050

**Published:** 2024-06-06

**Authors:** Ryoichiro Yamazaki, Junichi Ushiyama

**Affiliations:** ^1^Graduate School of Media and Governance, Keio University, Fujisawa, Japan; ^2^Faculty of Environment and Information Studies, Keio University, Fujisawa, Japan; ^3^Department of Rehabilitation Medicine, Keio University School of Medicine, Tokyo, Japan

**Keywords:** rhythm perception, auditory-motor coordination, syncopation, vestibular system, bimanual movements

## Abstract

Head movements that are synchronized with musical rhythms often emerge during musical activities, such as hip hop dance. Although such movements are known to affect the meter and pulse perception of complex auditory rhythms, no studies have investigated their contribution to the performance of sensorimotor synchronization (SMS). In the present study, participants listened to syncopated auditory rhythms and flexed their dominant hand index finger in time with the perceived pulses (4/4 meters). In the first experiment (Exp. 1), the participants moved their heads via voluntary neck flexion to the pulses in parallel with finger SMS (Nodding condition, ND). This performance was compared with finger SMS without nodding (Without Nodding condition, WN). In the second experiment (Exp. 2), we investigated the specificity of the effect of head SMS on finger SMS confirmed in Exp. 1 by asking participants to flex their bilateral index fingers to the pulses (Bimanual condition, BM). We compared the performance of dominant hand finger SMS between the BM and ND conditions. In Exp. 1, we found that dominant hand finger SMS was significantly more stable (smaller standard deviation of asynchrony) in the ND versus WN condition (*p* < 0.001). In Exp. 2, dominant hand finger SMS was significantly more accurate (smaller absolute value of asynchrony) in the ND versus BM condition (*p* = 0.037). In addition, the stability of dominant hand finger SMS was significantly correlated with the index of phase locking between the pulses and head SMS across participants in the ND condition (*r* = −0.85, *p* < 0.001). In contrast, the stability of dominant hand finger SMS was not significantly correlated with the index of phase locking between pulses and non-dominant hand finger SMS in the BM condition (*r* = −0.25, *p* = 0.86 after multiple comparison correction). These findings suggest that SMS modulation depends on the motor effectors simultaneously involved in synchronization: simultaneous head SMS stabilizes the timing of dominant hand finger SMS, while simultaneous non-dominant hand finger SMS deteriorates the timing accuracy of dominant hand finger SMS. The present study emphasizes the unique and crucial role of head movements in rhythmic behavior.

## 1 Introduction

People can move their hands or feet in time with the rhythm of music. Such movement is a type of sensorimotor synchronization (SMS; Repp, 2005), defined as repetitive movements synchronized in time with periodic perceptual events. In addition to hands or feet, head movements sometimes emerge simultaneously during musical activities such as playing musical instruments or hip hop dance. Do such head movements play any functional role in SMS in the hands or feet?

Considering that many previous studies have reported the advantage of bimanual SMS in synchronization stability over unimanual SMS (“bimanual advantage”; Pollok et al., 2007), it is reasonable to expect that simultaneous head movements could improve SMS in other body parts. As for bimanual advantage, there have been two major conventional hypotheses: the multiple-timer model hypothesis and the sensory feedback hypothesis (Studenka et al., 2018). In the multiple-timer model hypothesis, it is assumed that the two clock signals for two effectors are averaged before being sent to each effector, resulting in reduction of the variance of timing (Ivry and Richardson, 2002). On the other hand, in the sensory feedback hypothesis, additional sensory feedback for timing movements, e.g., touches or sounds of table tapping, is assumed to be beneficial to the reduced variance of timing movements (Drewing et al., 2002; Drewing and Aschersleben, 2003). Given these two hypotheses, head movements might improve SMS in the other effectors via the same mechanism as the bimanual advantage. However, no studies have investigated such a possibility.

On the contrary, it is also considered that head movements interact with SMS in a different manner from bimanual advantage because of a specific relationship between head movements and the meter and pulse perception of auditory rhythms. Phillips-Silver and Trainor (2005, 2007, 2008) demonstrated that the rhythm of head movements affected the perceptual judgment of auditory metrical structure. In these studies, the auditory meter for ambiguous metrical structure was perceived as identical to the rhythm of head movements experienced in the training. They also confirmed that the other body parts did not replicate the same results. Trainor et al. (2009) proved that this shifting modulation of auditory metrical perception was derived from the vestibular system by replacing head movements with galvanic vestibular stimulation (GVS). Recently, Tichko et al. (2021) succeeded in simulating this modulation with the neural-network model trained on combined auditory–vestibular stimulation. These findings support the specific role of head movements on meter and pulse perception. Considering that SMS is less stable when auditory rhythms are complex due to syncopation (Patel et al., 2005), head movements to syncopated rhythms are expected to help extract meters and pulses from complex metrical structures, which would contribute to SMS to such rhythms. This process would be different from bimanual advantage and specific to head movements eliciting vestibular signals. However, it has not been investigated whether SMS in the head improves SMS in the other body parts through the different mechanism of the bimanual advantage.

Thus, the primary purpose of the present study was to investigate whether SMS in the head improves that in other body parts. The first experiment (Exp. 1) compared the quality of SMS of the index finger of the dominant hand to auditory rhythms (i.e., the stability and accuracy of timing movements) in two different conditions. In the Nodding condition (ND), dominant hand finger flexion in time with the presented auditory rhythms was accompanied by head movements induced by voluntary neck flexion in time with the same rhythms. In the Without Nodding condition (WN), the dominant hand finger flexion was engaged in SMS to the auditory rhythms alone. In outline, Exp.1 revealed that the stability of SMS in the finger improved when simultaneously executed with SMS in the head. After obtaining this result, an additional goal arose: clarifying whether other effectors replicate such an impact of SMS in the head observed in Exp.1. The second experiment (Exp. 2) compared the quality of SMS in the ND condition to that in the Bimanual condition (BM), in which the participants were asked to flex both the index fingers on their dominant and non-dominant hand to the same auditory rhythms. Briefly, Exp.2 revealed that the parallel execution of SMS in the head and the dominant hand finger showed different performances from bimanual SMS. In both experiments, we used syncopated auditory sequences rather than isochronous metronomes to avoid a ceiling effect in which meter and pulse perception had no room for improvement. In addition, to examine the influences of the vestibular inputs from head movements without neural recordings as rigorously as possible, we designed the experiments so that participants flexed their fingers in the air with their eyes closed, minimizing the possibility of sensory feedback other than the vestibular one affecting the results.

## 2 Materials and methods

### 2.1 Ethical approval

All experiments were conducted in accordance with the Declaration of Helsinki except that the study was not pre-registered in a database and approved by the Research Ethics Committee at Shonan Fujisawa Campus, Keio University (receipt number 293). The participants received sufficient explanations about the experimental purpose and methods and provided written informed consent in advance of participation.

### 2.2 Participants

Thirty (21 women and 9 men; age 21.4 ± 2.92 years) and 33 (19 women and 14 men; 22.2 ± 2.84 years) healthy young adults participated in Exp. 1 and Exp. 2, respectively. Such a sample size was determined through both the survey of similar previous studies (e.g., Studenka et al., 2014) and computation by G*Power 3.1.9.4 (Faul et al., 2007) assuming the paired t-test (two-tailed, effect size of 0.5 to 0.6, power of 0.8 and alpha of 0.05). The following participants were excluded from the analyses: (1) those with a history of neurological disease or impaired auditory or motor function; (2) those judged by the experimenter to be unable to correctly synchronize their movements to the stimuli. The exclusion criterion for such participants showing incorrect synchronization depended on the consistency between the number of movements and that of pulses (i.e., 64): those who showed a larger or smaller number of finger flexion and head movements than that of pulses were excluded. Eventually, we analyzed data from 25 and 26 participants in Exp. 1 and Exp. 2, respectively. At the beginning of each experiment, we used the Japanese version of the Flinders Handedness survey (FLANDERS; Nicholls et al., 2013; Okubo et al., 2014) to assess the handedness of each participant. It revealed that 23 and 24 participants were right-handed in Exp. 1 and Exp. 2, respectively.

## 3 Procedure

### 3.1 Auditory stimuli

The auditory stimuli consisted of an isochronous countdown and a repeated rhythmic pattern. The rhythmic patterns were the 10 syncopation patterns (Table 1) used in Chapin et al. (2010). Note that we used all of the 10 patterns because we had no criteria for evaluate which of them was proper for our experiments or not. Each pattern comprised 2 bars in 4/4 time, where 4 tones were placed at the quarter-note level (i.e., onbeats or strong beats) and the other 4 tones at the eighth-note level (i.e., offbeats or week beats). Such patterns were reported to induce pulse perception at the quarter-note level (Chapin et al., 2010). In each trial, we first presented a countdown comprising 4 tones, and then a rhythmic pattern that repeated 8 times as a continuous sequence. The rhythmic pattern stimuli were composed of 440-Hz pure tones and the countdown stimuli were 220-Hz pure tones. Each tone lasted 50 ms and had a steady-state duration of 30 ms, a 10-ms rise and fall time (linear ramps), and a sampling frequency of 44.1 kHz. The tones were presented in 4/4 time at 110 beats per minute, i.e., the intervals between 4/4 meters (perceived pulses) were about 545 ms. We presented each stimulus with white noise to reduce the influence of acoustic noise from the surrounding environment. The amplitude of the white noise rose linearly starting at the second countdown tone and reached the same intensity as the stimulus tones at the second 4/4 meter of the rhythmic pattern. This white noise fade-in ensured that the participants clearly heard the first countdown tone. This procedure for noise cancelation was based on previous studies (Elliott et al., 2010; Roy et al., 2017) and established through our pilot experiments. In addition to these stimuli, we created 10 additional “less syncopated” sequences, i.e., sequences containing more onbeats and fewer offbeats, for the training session before the experiment.

**Table 1:** The syncopated auditory rhythm patterns used in the experiments.

Pattern																
4/4	X	–	X	–	X	–	X	–	X	–	X	–	X	–	X	–
#1	X	–	–	**/**	X	–	X	–	–	**/**	X	**/**	–	**/**	–	–
#2	X	**/**	–	**/**	–	–	X	**/**	–	**/**	X	–	X	–	–	–
#3	X	**/**	X	–	X	**/**	–	**/**	–	–	–	**/**	–	–	X	–
#4	X	–	–	**/**	–	**/**	–	**/**	–	–	X	–	X	**/**	X	–
#5	X	–	X	–	–	**/**	–	**/**	X	–	–	**/**	–	**/**	X	–
#6	X	**/**	X	**/**	–	–	X	–	–	**/**	–	**/**	X	–	–	–
#7	X	–	–	**/**	–	–	–	–	X	**/**	X	**/**	–	**/**	X	–
#8	X	–	X	–	–	**/**	–	**/**	X	**/**	X	**/**	–	–	–	–
#9	X	**/**	X	–	–	**/**	X	**/**	–	–	–	**/**	X	–	–	–
#10	X	–	X	**/**	–	**/**	–	–	X	**/**	–	**/**	–	–	X	–

All patterns were the replication of ones in Chapin et al. (2010). “X” represents onbeat tones while “/” represents offbeat ones. “–” indicates the absence of tones (i.e., silence). Each sequence comprised 2 bars in 4/4 time, which was continuously looped 8 times in each trial. The top row illustrates the isochronous (unsyncopated) sequence of 4/4 meters, showing the timing of perceived pulses to be synchronized.

### 3.2 Experimental trials

The participants sat comfortably on a specialized chair. The height of its armrests was adjustable at 3 levels, and this height was adjusted for each participant so that their forearms lay horizontal on the armrests. The metacarpophalangeal (MP) joints in the hand used in the tasks were not touching the armrests. Each participant’s index finger (on the task hand) was secured in an extended position via tape attached from the MP joint to the nail. Before the beginning of the experiment, the volume of the auditory stimuli was set to the loudest comfortable level, ensuring that the participants could hear the tones clearly.

Before the experiment, the participants completed a practice session including finger tapping, finger flexion, nodding, and the parallel execution of finger flexion and nodding in time with the stimuli. All practiced movements were synchronized with the pulses of the auditory rhythms. In the first step of the practice session, the participants practiced tapping the surface of a horizontal platform placed under their hand with the index finger of their dominant hand. The height of the platform was adjusted so that the participant’s fingertip touched the platform surface when the MP joint was flexed at approximately 45 degrees. The participants practiced quick finger flexion at the MP joint to learn the required angular displacement for flexion. In the second step, the participants practiced finger flexion in the air, i.e., without the platform, with the same degrees of flexion as in the previous step. The purpose of these first and second steps was to ensure that participants recognized the timing when the fingertip reached the lowest position during flexion without tactile contact as the timing to be synchronized with the stimuli. In the third step, they practiced SMS in the head by nodding their head downward to the pulses to a comfortable degree such that they felt no pain and minimal fatigue. Note that, we did not train participants to recognize a particular timing as the target for synchronization in head movements because we had to minimize the effort for head movements (i.e., attentional demands toward the head movements), which would likely deteriorate the performance of finger flexion. Finally, in the last step of the practice session, they practiced the parallel execution of finger flexion and nodding. They were instructed to avoid coordinating these movements with each other, and to independently synchronize each movement to the perceived pulses. The purpose of such instruction was to prevent participants from executing the task as a primary auditory-motor coupling and a secondary motor-motor coupling and to lead them into executing it as two parallel auditory-motor couplings. In other words, the instruction prohibited participants from recognizing finger flexion as the cue for head movements and vice versa. In Exp. 2, the practice of bimanual finger flexion, the same SMS as the task in the BM condition, was added to this last step.

The participants listened to different auditory sequences in each trial. The order of the rhythmic sequences was fixed so that the less syncopated sequences (i.e., more onbeats and fewer offbeats) were presented in the earlier trials. This order was set to ensure that the participants got accustomed to the synchronization to the pulses as the degree of syncopation increased. The participants could repeat any of the above steps until they felt sufficiently accustomed to the movements. Each step consisted of 2 trials. The duration of each trial was identical to that in the following experiment, approximately 40 s. The duration of practice was about 15 min on average. At the beginning of the practice session, the experimenter demonstrated the tapping technique. During the following practice trials, the participants kept their eyes closed.

In Exp. 1, each participant performed sensorimotor synchronization tasks in 2 conditions, i.e., the ND and WN conditions. In the ND condition, as in the last step of the practice session, the participant flexed the index finger on their dominant hand to the pules in parallel with downward nodding movements, generated to synchronize with the same pulse. In the WN condition, the participant did not nod their head, but just synchronized the dominant hand finger flexion in the air to the pulses, as in the second step of the practice session. In Exp. 2, in addition to the ND condition, the participants were presented with the BM condition, in which they were expected to flex both index fingers in the air to the pulses. During the experimental trials, the participants sat up straight in the chair with their backs not in contact with the backrest, and they were blindfolded with a sleep mask to minimize any interference from visual or tactile information. In the WN and BM conditions, they wore a U-shaped cushion around their neck to prevent spontaneous head movements and reduce the attentional demand to suppress such movements as possible. In both experiments, they were instructed not to count the number of tones and not to mentally replace the presented rhythms with any words or melodies. Note that all instructions provided to participants were identical between the experiments.

We provided the following instructions to participants. “Please start tapping (finger flexion or head nodding in the subsequent practices or tasks) to four lower-pitched countdown tones as soon as you hear the first one. These tones indicate the 4/4 meters, so-called beats. Although the subsequent higher-pitched tones will form a rhythm pattern and will not always have regular intervals between each other, please keep tapping (finger flexion or head nodding) synchronized to the 4/4 meters. That is, you will move your finger (or head) like a metronome or as you clap to musical beats in a live concert.” In other words, the participants were asked to begin synchronizing to the countdown tones, which indicated the 4/4 meters of the following syncopated rhythm, immediately when they heard them and to keep synchronizing to the perceived pulses until the stimulus ended. Each of the 10 syncopation patterns was presented once for each condition (ND and WN in Exp. 1; ND and BM in Exp. 2) so that each participant completed 20 trials overall. The order of the 2 conditions and the 10 syncopation patterns was quasi-randomized within and across participants. In Exp. 1, each of the 10 syncopation patterns was randomly assigned to 1 of the first 10 trials. This pattern was repeated in the last 10 trials. Each of the 2 conditions was randomly assigned within the first 10 trials so that the conditions were presented equally (5 trials each). This randomization was reversed in the last 10 trials (e.g., the 11th trial was ND when the first trial was WN). This procedure was set so that each condition had the same stimulus order effect. In Exp. 2, the order of the ND and BM conditions could not be randomized because of the time needed to attach the sensors when switching conditions. Therefore, each condition was randomly assigned to the first or last 10 trials across the participants, and the order of the syncopation pattern was randomized in the same manner as in Exp. 1. In both experiments, the condition of the first trial was balanced across participants. Note that the differences between the WN condition in Exp. 1 and the BM condition in Exp. 2 were the employment of the non-dominant hand and the blocked design. The participants could request a break of several minutes anytime during the trial session. In both experiments, all participants took at least 1 break. The total experimental session, from the first trial after practice to the last one, lasted approximately 1 hour.

## 4 Apparatus and data collection

We used wireless inertial sensors (WaveTrack Inertial System, Cometa Systems, Milan, Italy) to record the angular displacements at a sampling frequency of 140 Hz. Two sensors were attached to the dorsal surface of the dominant hand (i.e., 1 over the second metacarpal bone and 1 over the second proximal phalanx of the dominant hand). They were placed in a line so that the second MP joint was in the middle of the sensors. We used 2 additional sensors to record the angular displacement of the neck joint in the ND condition (Exp. 1 and 2) or the second MP joint in the non-dominant hand in the BM condition (Exp. 2). In the ND condition, 1 of the sensors was attached to the center of the participant’s forehead, and the other was placed on the skin over the posterior aspect of the spinous process of the seventh cervical vertebra. In the BM condition, the 2 sensors were attached to the non-dominant hand in the same manner as the dominant hand.

We used 3 computers to present the stimuli and record the data. The recorded data were saved on the first laptop (G-Tune E5-CLR, Mouse Computer Co., Ltd., Tokyo, Japan) via EMG and Motion Tools processing software (Version 7.4.6.0, Cometa Systems, Milan, Italy). We used MATLAB software (The Mathworks Inc., MA, United States, Version 9.8.0.1451342, R2020a) and Psychtoolbox 3.0.18 for MATLAB (Brainard, 1997) on the second laptop (MacBook Pro; 13-inch, 2017, Apple Inc., CA, USA) to produce and present the auditory stimuli. The auditory stimuli were presented through headphones (MDR-7506, Sony Corporation, Tokyo, Japan). Concurrently, the stimulus was inputted into an analog-to-digital converter (USB-6212 (BNC), National Instruments Corporation, TX, USA), converted to a digital signal, and saved on the third laptop (G-Tune NEXTGEAR-NOTE W656RC, Mouse Computer Co., Ltd., Tokyo, Japan) with a sampling frequency of 1,000 Hz. This A/D converter realized the temporal synchronization between these 3 computers. At the beginning of each trial, the third computer initiated this converter, immediately sending a trigger signal to the first computer for kinematic data. Meanwhile, the analog signals of auditory stimuli generated by the second computer were directory input into the converter. This technique was independent of delays in computer processing times, guaranteeing precise temporal synchronization between the auditory stimuli and the kinematic data.

## 5 Data analyses

**Figure 1:**
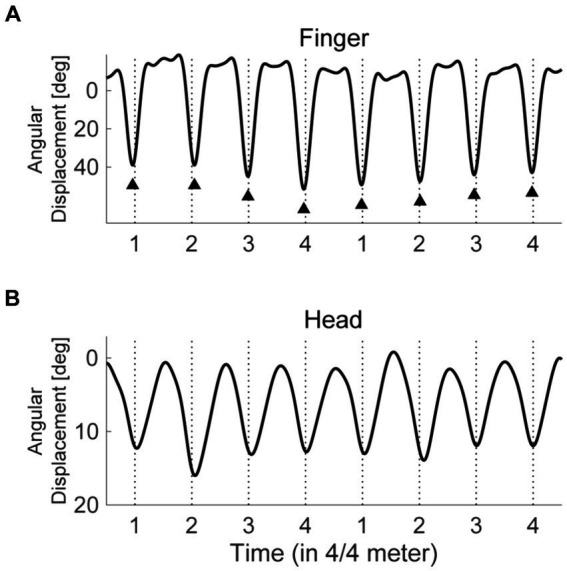
The trajectories of finger and head movements. The solid lines represent the angular displacement of finger flexion **(A)** and neck flexion **(B)** for a typical participant. The flexion angle exhibits 0 when the finger or neck stretches. The vertical broken lines depict the timings of 4/4 meters (perceived pulses). The triangle markers indicate the detected local peaks of angular displacement, which are defined as the onsets of finger flexion.

The angular displacements were low-pass filtered at 6 Hz with a fourth-order Butterworth filter. We used the “findpeaks” function in MATLAB to extract the prominent peaks of the displacements closest to each onset of the 4/4 meter for the finger flexion in the dominant hand (Figure 1). The extracted peaks were defined as the onsets of the synchronized movements. Before the quantitative analyses, we visually confirmed that this process correctly detected the onsets. When such visual inspection found errors for onset detection on rare occasions, we adjusted the parameters in the codes so that they successfully detected the onsets. For each trial, we obtained the onsets of finger flexion to 64 pulses in the syncopated rhythms, and we discarded those to the preceding 4 countdown tones. To evaluate the SMS performance, we defined the asynchrony of synchronization (ASY) as the temporal distance between the onset of a 4/4 meter (perceived pulse) and the maximal angular displacement of the dominant hand finger flexion closest to the meter. It is to be noted that although the timing of maximal angular displacement is not frequently adopted as the onset of SMS in the finger, the calculated ASY (Supplementary Tables S1, S4) did not differ considerably from the values reported in previous studies using usual tapping tasks [e.g., Aschersleben et al., 2001]. The absolute value of ASY (ASY_abs_) was calculated to evaluate the accuracy of synchronization, while the standard deviation of ASY (ASY_SD_) was calculated to evaluate the stability of synchronization. Smaller values of ASY_abs_ or ASY_SD_ reflected more accurate and stabler synchronization, respectively.

Given that head SMS likely enhanced meter and pulse perception, we expected the quality of head SMS to be associated with the dominant hand SMS. However, it is unable to define and calculate the onsets of head movements due to their smooth continuous trajectory (Figure 1). As well, it was unclear whether each participant recognized the timing when the heads reached the lowest position as the target for head movements. Therefore, we calculated the movement phase relative to the pulses or dominant hand finger SMS for the head (Exp. 1 and 2) and non-dominant hand index finger (Exp. 2), which did not require asynchronies for evaluating the stability of SMS. The calculation was based on previous studies (Phillips-Silver et al., 2011; Fujii et al., 2014). The vertical acceleration data obtained from the inertial sensors on the fingers and forehead were bandpass filtered using a zero-phase fast Fourier transform filter with a center frequency of 1.83 Hz, equal to the 4/4 meters, and a bandwidth of 20% of this frequency. We then used the Hilbert transform to obtain the instantaneous phase of the filtered signal. The relative phase between the pulse and the finger flexion or head movements was defined by subtracting the instantaneous phase for the finger flexion or head movement from that for the 4/4 meter. Note that the instantaneous phase of the 4/4 meter was defined as “a linear increase from −180 to 180 degrees between the beat onsets” (Fujii et al., 2014). Similarly, we calculated the relative phases between head movements and the dominant hand finger flexion or between the finger flexion in both hands. We then calculated the circular mean of this relative phase. We used the length of the mean vector as an index of the stability of synchronization or phase locking, referred to hereafter as the phase locking index (PLI; 1 represents an absence of variability while 0 represents maximum variability; Large et al., 2015). The circular mean of the relative phases served as an evaluation of the stability of synchronization in the head or non-dominant hand. Note that we adopted vertical acceleration instead of angular displacement for PLI calculation due to the property of the Hilbert transform, which is a stationary process (Gatto, 2022). As well, the acceleration data was segmented from 545 ms before the first tone (i.e., from the timing of the last countdown tone) to 545 ms after the last one for the bandpass filter, and these buffers of 545 ms were discarded after the Hilbert transform, canceling the influences of the skews in the edges of Hilbert data (Pikovsky et al., 2001).

## 6 Statistical analyses

Each of the indices, ASY, ASY_SD_, and ASY_abs_, was averaged within each participant. Before conducting statistical comparisons, we used the MATLAB function “isoutlier” to detect the participants with outlier data for each of these indices. Specifically, we used the input argument “quartiles” for the “Method” parameter. “Outliers are defined as elements more than 1.5 interquartile ranges above the upper quartile (75 percent) or below the lower quartile (25 percent)” (“Find outliers in data - MATLAB isoutlier,” https://www.mathworks.com/help/matlab/ref/isoutlier.html, last accessed on September 23rd, 2023). Participants with outliers for at least one of the indices were discarded from the subsequent analyses. Consequently, 25 participants remained in each Exp. 1 and Exp. 2.

Before comparing the data between the 2 conditions, we used the Shapiro–Wilk test to check the normality of the distribution for each obtained dataset. Bartlett’s test and Levene’s test checked the homogeneity of the data variance given a normal or non-normal distribution, respectively. Because these tests revealed violations of either homogeneity or normality for each dataset, we used the two-tailed Wilcoxon signed rank test to check the statistical differences between the data for the different conditions. The behavioral indices showing significant differences between conditions were used to examine the correlation with the PLI. We calculated the PLI between the head SMS and the perceived pulses (PLI_Head-Pulse_) or the dominant finger SMS (PLI_Head-Dom_) for the ND condition in both Exp. 1 and 2. We also calculated the PLI between the non-dominant finger SMS and the pulse (PLI_Non-Pulse_) or the dominant finger SMS (PLI_Non-Dom_) for the BM condition in Exp. 2. Participants with outliers in the PLI were detected in the aforementioned manner and discarded from the correlational analysis. Consequently, 21 participants remained in Exp. 1 and 20 remained in Exp. 2. Pearson correlations were calculated across these participants. We used the Benjamini-Hochberg method to correct the false discovery rate (FDR) for the multiple correlational analyses (2 tests included for the ND condition in Exp. 1, and 4 per condition in Exp. 2; Benjamini and Hochberg, 1995). The corrected *p*-values are reported as *p*_FDR_ in this article. The level of significance was set at 0.05. Since the present tasks were unusual in syncopated stimuli and SMS without tactile feedback, sensitivity analyses with the outliers (Thabane et al., 2013) were performed for all statistical tests above, which enables future researches to investigate the robustness of the exclusion of outlier data.

## 7 Results

### 7.1 Exp. 1

#### 7.1.1 Descriptive statistics

**Figure 2:**
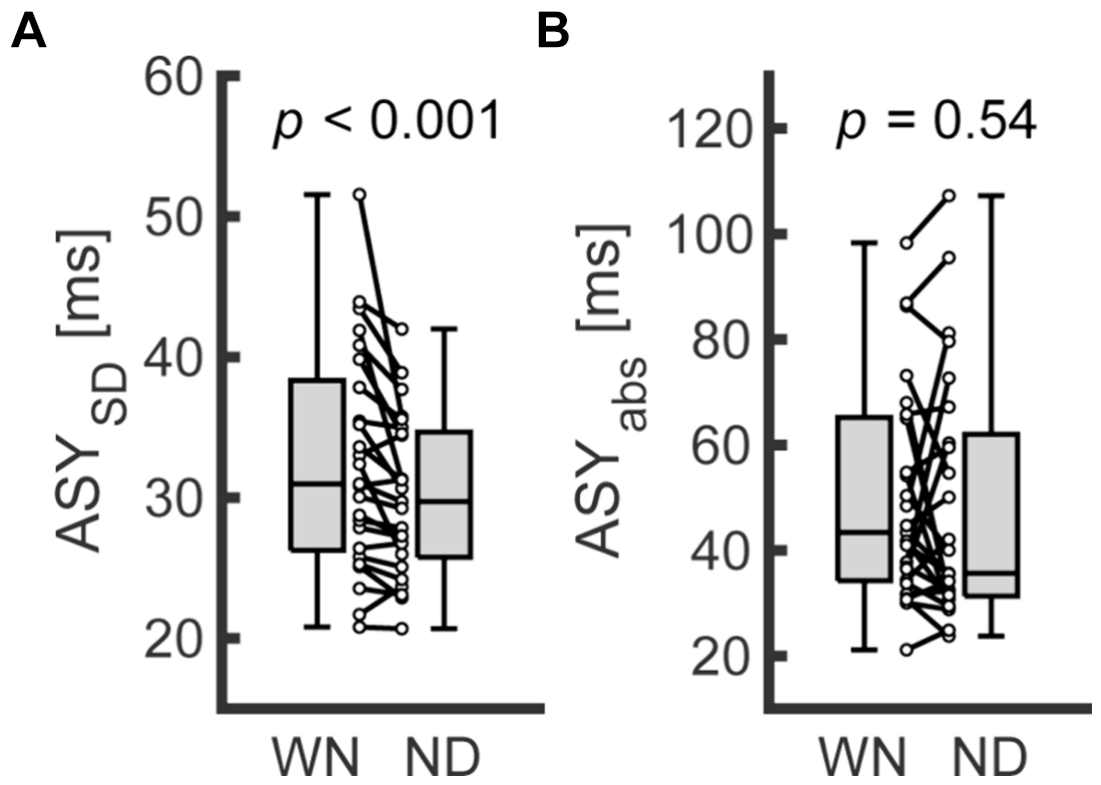
Comparison of behavioral indices between conditions in Exp. 1. **(A,B)** represent the standard deviation of asynchrony (ASY_SD_, left) and the absolute values of asynchrony (ASY_abs_, right) for finger SMS, respectively. Each dot represents data from each participant. Boxplots represent the median, the first/third quartile, and the minimum/maximum values in the center line, box limits, and whiskers, respectively. WN, without nodding condition; ND, nodding condition.

Supplementary Table S1 summarizes the descriptive data from Exp. 1. The ASY tended to shift toward a positive value in the ND condition (−22.67 and −32.46 for the ND and WN conditions on average, respectively). However, the difference in the mean ASY did not differ between the 2 conditions (*n* = 25; Wilcoxon signed rank test; *W* = 92, *z* = −1.90, *p* = 0.058). In contrast, the ASY_SD_ was significantly smaller in the ND condition compared with the WN condition (Figure 2A; *n* = 25; Wilcoxon signed rank test; *W* = 293, *z* = 3.51, *p* < 0.001). The ASY_abs_ was not different between the conditions (Figure 2B; *n* = 25, Wilcoxon signed rank test; *W* = 185, *z* = 0.605, *p* = 0.54).

#### 7.1.2 Correlational analysis

**Figure 3:**
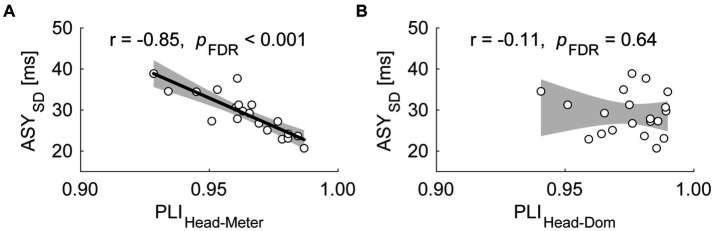
Scatter plots of the standard deviation of asynchrony (ASY_SD_) against the phase locking index (PLI) between the head SMS and the perceived pulses [PLI_Head-Pulse_, **(A)**], as well as the PLI between the head SMS and dominant hand finger SMS [PLI_Head-Dom_, **(B)**] in Exp. 1. Each dot represents each participant. The shaded area corresponds to the confidence interval of the r value. The thick line indicates the linear regression.

We used Pearson correlations to evaluate the relationship between the quality of dominant finger SMS and that of head SMS. Supplementary Table S2 summarizes the results of the correlational analyses. In the ND condition, the PLI_Head-Pulse_ was significantly negatively correlated with the ASY_SD_ (Figure 3; *r* = −0.85; the confidence interval (CI) of *r* = [−0.94, −0.66]; *p*_FDR_ < 0.001). In other words, participants showing stabler head SMS also showed stabler dominant hand finger SMS. Meanwhile, the PLI_Head-Dom_ was not significantly correlated with the ASY_SD_ [*r* = −0.11; CI of *r* = (−0.53, 0.35); *p*_FDR_ = 0.64]. In the sensitivity analysis with outliers, we observed a similar significant correlation between the PLI_Head-Pulse_ and ASY_SD_ [Supplementary Table S3; *r* = −0.86; the CI of *r* = (−0.93, −0.70); *p*_FDR_ < 0.001]. Also, the analysis did not reveal a significant correlation between PLI_Head-Dom_ and ASY_SD_ [*r* = −0.11; CI of *r* = (−0.49, 0.30); *p*_FDR_ = 0.59].

### 7.2 Exp. 2

#### 7.2.1 Descriptive analysis

**Figure 4:**
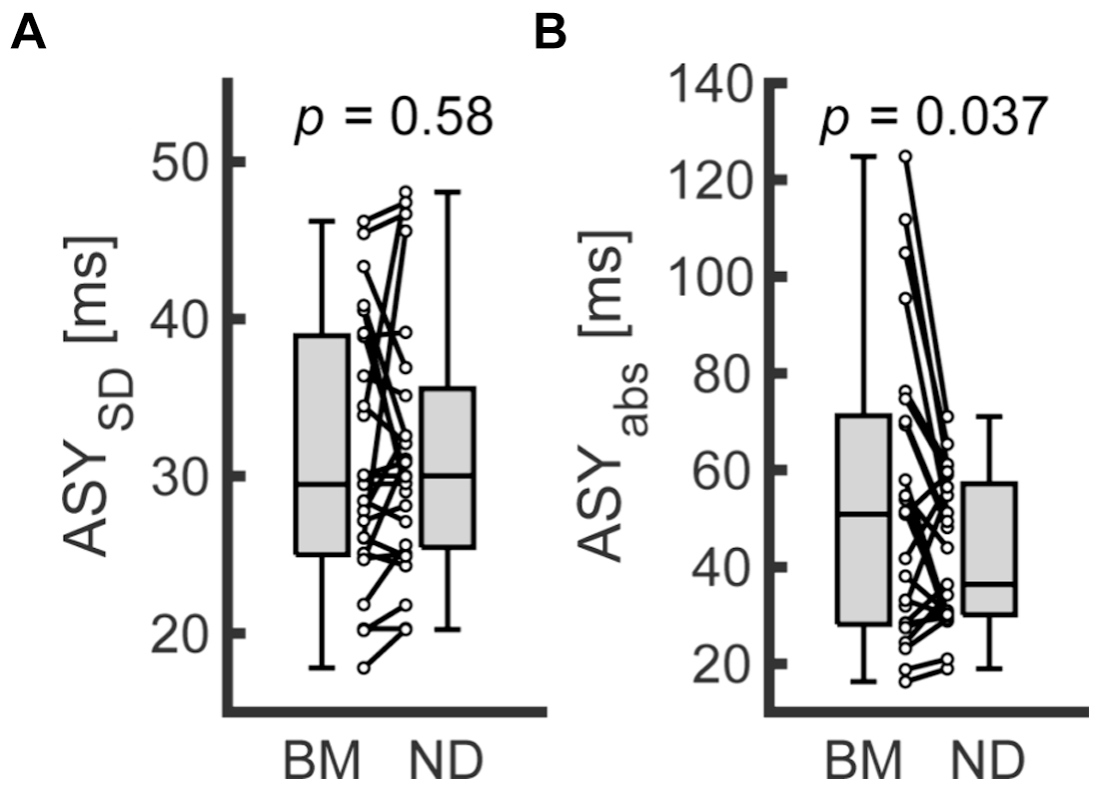
Comparison of behavioral indices between conditions in Exp. 2. **(A,B)** represent the standard deviation of asynchrony (ASY_SD_) and the absolute values of asynchrony (ASY_abs_) for finger SMS, respectively. Each dot represents each participant. Boxplots represent the median, the first/third quartile, and the minimum/maximum values in the center line, box limits, and whiskers, respectively. BM, bimanual condition; ND, nodding condition.

Supplementary Table S4 summarizes the descriptive data from Exp. 2. The mean ASY tended to shift toward a positive value in the ND condition (−24.74 and −36.75 for the ND and BM conditions on average, respectively). Nevertheless, it did not differ significantly from that in the BM condition (*n* = 25; Wilcoxon signed rank test; *W* = 95, *z* = −1.82, *p* = 0.069). Also, the ASY_SD_ did not differ significantly between the 2 conditions (Figure 4A; *n* = 25; Wilcoxon signed rank test; *W* = 142, *z* = −0.552, *p* = 0.58). However, the ASY_abs_ was significantly smaller in the ND condition compared with the BM condition (Figure 4B; *n* = 25; Wilcoxon signed rank test; *W* = 240, *z* = 2.09, *p* = 0.037). The results of the sensitivity analysis with outliers were inconsistent with those reported above: the differences between the conditions did not reach a significant level in terms of the ASY_abs_ (Supplementary Table S5; *n* = 26; Wilcoxon signed rank test; ASY: *W* = 109, *z* = −1.69, *p* = 0.091; ASY_SD_: *W* = 155, *z* = −0.521, *p* = 0.60; ASY_abs_: *W* = 251, *z* = 1.92, *p* = 0.055).

#### 7.2.2 Correlational analyses

**Figure 5:**
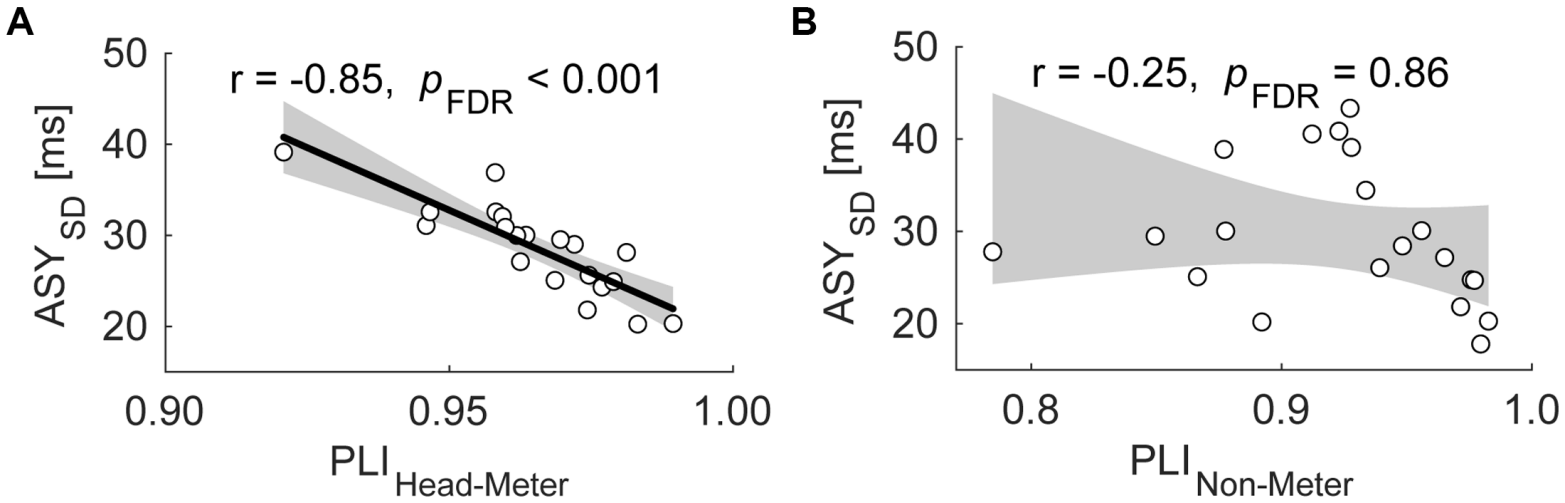
Scatter plots of the standard deviation of asynchrony (ASY_SD_) against the phase locking index (PLI) between the head SMS and the perceived pulses in the ND condition [PLI_Head-Pulse_, **(A)**], and the PLI between the non-dominant hand finger SMS and the pulses in the BM condition [PLI_Non-Pulse_, **(B)**] in Exp. 2. Each dot represents each participant. The shaded areas correspond to the confidence intervals of the *r* value. The thick line indicates the linear regression.

Supplementary Table S6 summarizes the results of the correlational analyses. Similar to Exp. 1, the PLI_Head-Pulse_ showed a significant negative correlation with the ASY_SD_ across participants [Figure 5A; *r* = −0.85; CI of *r* = (−0.94, −0.65); *p*_FDR_ < 0.001]. No significant correlations were observed between other PLI and behavioral measure pairs [PLI_Head-Pulse_ vs. ASY_abs_, *r* = −0.21; CI of *r* = (−0.59, 0.26); *p*_FDR_ = 0.46; PLI_Head-Dom_ vs. ASY_SD_, *r* = −0.44; CI of *r* = (−0.74, 0.00); *p*_FDR_ = 0.15; PLI_Head-Dom_ vs. ASY_abs_, *r* = −0.29; CI of *r* = (−0.65, 0.17); *p*_FDR_ = 0.31]. In the BM condition, we found no significant correlations between the PLI and behavioral measurements [PLI_Non-Pulse_ vs. ASY_SD_, *r* = −0.25; CI of *r* = (−0.62, 0.22); *p*_FDR_ = 0.86 (Figure 5B); PLI_Non-Pulse_ vs. ASY_abs_, *r* = −0.25; CI of *r* = (−0.62, 0.22); *p*_FDR_ = 0.58; PLI_Non-Dom_ vs. ASY_SD_, *r* = 0.19; CI of *r* = (−0.28, 0.58); *p*_FDR_ = 0.64; PLI_Non-Dom_ vs. ASY_abs_, *r* = 0.14; CI of *r* = (−0.32, 0.55); *p*_FDR_ = 0.67].

The sensitivity analyses with outliers revealed significant correlations between the PLI_Head-Pulse_ and ASY_SD_ [Supplementary Table S7; *r* = −0.88; CI of *r* = (−0.94, −0.74); *p*_FDR_ < 0.001]. No significant correlations were observed between other PLI and behavioral measurement pairs [PLI_Head-Pulse_ vs. ASY_abs_, *r* = −0.36; CI of *r* = (−0.66, 0.03); *p*_FDR_ = 0.11; PLI_Head-Dom_ vs. ASY_SD_, *r* = −0.30; CI of *r* = (−0.62, 0.10); *p*_FDR_ = 0.16; PLI_Head-Dom_ vs. ASY_ABS_, *r* = −0.40; CI of *r* = (−0.68, −0.02); *p*_FDR_ = 0.08; PLI_Non-Pulse_ vs. ASY_SD_, *r* = −0.23; CI of *r* = (−0.56, 0.18); *p*_FDR_ > 0.27; PLI_Non-Pulse_ vs. ASY_abs_, *r* = −0.17; CI of *r* = (−0.52, 0.24); *p*_FDR_ > 0.41; PLI_Non-Dom_ vs. ASY_SD_, *r* = 0.04; CI of *r* = (−0.35, 0.42); *p*_FDR_ > 0.85; PLI_Non-Dom_ vs. ASY_abs_, *r* = −0.04; CI of *r* = (−0.42, 0.35); *p*_FDR_ > 0.85].

## 8 Discussion

### 8.1 Exp. 1 – Head movements stabilized finger SMS

In Exp. 1, we found a smaller ASY_SD_ in the ND versus WN condition (Figure 2A), indicating that simultaneous execution of head SMS reduced the timing variability of finger SMS. In the ND condition, we expected head movements to provide additional feedback from proprioception and the vestibular system, and to increase the number of feedback sources. Indeed, previous studies have indicated that such additional feedback might have improved the timing stability of finger flexion (Studenka et al., 2021). As for proprioception, Bravi et al. (2017) reported that reinforcing proprioceptive feedback via the application of an elastic therapeutic device reduced the timing variability of rhythmic finger flexion. Given the abundance of muscle spindles in neck muscles (Banks, 2006; Kissane et al., 2023), rich proprioceptive information from neck flexion might serve as a secondary feedback source, improving the stability of finger SMS.

In addition to proprioception, vestibular feedback from head movements may function as an additional sensory source. For instance, the rhythm of vestibular feedback is likely crucial for SMS. Trainor et al. (2009) reported that the perceptual judgment of an auditory metrical structure was biased toward the rhythm of vestibular stimulation. In light of this previous finding, the meter and pulse perception for syncopated rhythms might be more precise when head movements are synchronized to the pulses, leading to better SMS performance. Hence, we used phase analyses to confirm the relationships between SMS precision in the head and finger in the ND condition. We found that the PLI_Head-Pulse_ was negatively correlated with the ASY_SD_ in the ND condition (Figure 3A). In other words, the participants with more stable head synchronization to the pulses also showed more stable finger SMS timing to the same pulses. However, the results obtained in the ND condition might not have been specific to head movements, but instead reflected the simultaneous use of multiple body parts. To address these issues in Exp. 2, we employed the non-dominant hand index finger in the BM condition as a counterpart to the head in the ND condition.

### 8.2 Exp. 2 – Head movements affect SMS via a different mechanism from the non-dominant hand

In Exp. 2, we found that the ASY_abs_ was larger in the BM versus ND condition (Figure 4B). This indicates that, compared with the addition of the SMS in the non-dominant finger, the addition of the SMS in the head was beneficial to the SMS in the dominant hand finger. Indeed, we could not directly compare the performance between the BM and WN conditions because we did not include them in the same experiment. However, considering that we found no significant difference in ASY_abs_ between the ND and WN conditions in Exp. 1, the timing accuracy in the BM condition in Exp. 2 could be lower than in the WN condition. Note that the present finding for the BM condition was not necessarily inconsistent with the previous findings of bimanual advantage because most previous studies have reported the advantage of bimanual SMS in its stability (Helmuth and Ivry, 1996; Drewing et al., 2002, 2004). The potential decrease in accuracy in the BM condition was likely related to the use of the non-dominant hand. Fitch and Rosenfeld (2007) reported that the timing accuracy decreased in proportion to the degree of syncopation. In addition, Witek et al. (2014) inferred that syncopation in strong metrical positions induces a less stable perception of rhythmic patterns and greater asynchronies compared with that in weak metrical positions. Considering these findings, it is likely that the use of the non-dominant hand led to deteriorated meter and pulse perception for stimuli with strong syncopation, leading to more inaccurate timing in the BM condition than in the ND condition.

Phase analysis confirmed that the PLI_Non-Pulse_ and PLI_Non-Dom_ were not correlated with any behavioral indices of the dominant hand finger SMS in the BM condition (Figure 5B). The absence of a correlation suggests that the SMS quality in the dominant hand in the BM condition was independent of the SMS quality in the non-dominant hand. Meanwhile, similar to Exp. 1, the PLI_Head-Pulse_ was negatively correlated with the ASY_SD_ in the ND condition. In other words, unlike the SMS in the non-dominant hand, the quality of the SMS in the head was associated with that in the dominant hand. Even though we did not record the actual neural activity, this incongruence between the two conditions presumably suggests that the parallel execution of head SMS and dominant hand SMS reduced the ASY_SD_ via a different neural mechanism compared with bimanual SMS, which has been reported to cause the timing stabilization of SMS (Helmuth and Ivry, 1996; Pollok et al., 2007; Studenka et al., 2018). Overall, Exp. 2 revealed the specific and positive impact of head movements on simultaneous SMS.

## 9 General discussion

In the present study, we obtained the following main findings: (1) the stability of SMS in the dominant hand index finger to syncopated auditory rhythms was stabler when head SMS was simultaneously executed to the same rhythms; (2) SMS in the dominant hand finger was similarly stable but less accurate when executed simultaneously with SMS in the non-dominant hand index finger than when with that in the head; (3) the stability of head SMS to the perceived pulses was correlated with that in the dominant hand finger, while the stability of SMS in the non-dominant hand finger was not correlated with that in the dominant hand finger. Considering (1) and (2) together, although the two experiments differed in participants and block design, the stability of SMS in the dominant hand index finger in the ND condition was similar to that in the BM condition and higher than that in the WN condition. This suggests that the addition of an effector stabilizes the movement timings of SMS. Moreover, taking (3) into account, the neural mechanism stabilizing SMS in the dominant hand appears to differ between body parts engaged in simultaneous movements (i.e., the head or the non-dominant hand).

In the ND condition, we observed the correlation in timing stability between the finger flexion and head movements to the pulses. There are several potential interpretations for such a correlation. One is that it represents the confounder between the two groups of data. As a potential confounder, individual differences in SMS ability may explain this correlation. Indeed, recent studies demonstrated that experienced street dancers exhibit stable SMS in terms of both finger flexion and knee bending movements (Miura et al., 2013, 2016). These previous studies support the potential role of individual skill in SMS as a confounding variable, which could affect rhythmic movements concurrently executed in multiple body parts, such as those in the ND and BM conditions. However, the SMS timing stability in the dominant hand to the pulses was not correlated with that in the non-dominant hand in the BM condition. Thus, it is plausible that the individual SMS ability was not a confounder for the correlation between the stability of the finger and the head in the ND condition.

The stability correlation between the finger and the head without a confounder implies the directional effect of stabler head movements on stabler finger flexion and/or vice versa. Considering the reduced timing variability in the finger in the ND compared with the WN condition, such a correlation probably reflects that the stability in head movements affected that in the finger. The perceptual bias of judging auditory metrical structures toward the rhythm of head movements (Phillips-Silver and Trainor, 2005, 2007, 2008) likely explains such an effect. In other words, more precise head synchronization to the perceived pulses might induce more precise perception of the stimulus rhythm, leading to precise synchronization of the finger. On the other hand, owing to the shortage of previous studies focusing on SMS in the head, it is difficult to discuss the possibility of SMS in the finger affecting that in the head. Altogether, our correlational analysis of timing stability suggests a positive impact of concurrent head movements on precise SMS in the finger.

The potential role of head movements in improving SMS is supported from a neurological perspective. Head movements are represented and trigger responses in the internal segment of the globus pallidus (globus pallidus internus, GPi) and the substantia nigra par reticulata (SNr) of the basal ganglia (Joseph and Boussaoud, 1985; Hamada et al., 1990; Nambu, 2011; Todd and Lee, 2015; Horn et al., 2022). These subcortical substrates are known to compose the neural circuit involved in generating rhythmic movements (Galvan and Wichmann, 2008; Fujii and Wan, 2014). For example, deep brain stimulation targeting the GPi helps patients with movement disorders such as Parkinson’s disease execute smooth rhythmic movements (Miocinovic et al., 2013). Therefore, activities of the GPi or SNr triggered by head movements might interact with the neural circuit involved in generating rhythmic movements. In addition, some recent studies support the interaction of the vestibular system with the perception of auditory rhythms and the generation of rhythmic movement. Rocha et al. (2021a,b) revealed that the tempo of vestibular inputs derived from caregivers’ gait determined the tempo of spontaneous movements in their infants. Also, Tichko et al. (2021) succeeded in simulating the preference for auditory rhythms in infants with the neural-network model trained on combined auditory–vestibular stimulation. Considering these things, head movements and associated vestibular feedback might improve SMS in the finger.

We observed lower accuracy in the BM condition than in the ND condition. In addition, as shown in Figure 4B, some participants showed a considerable intra-individual decline in the accuracy for the BM condition qualitatively compared to the ND condition, a phenomenon reported in a minimal number of studies. Such studies are homogeneous in demonstrating inaccurate SMS in tasks requiring cognitive efforts such as multitasking or synchronizing to syncopated rhythms (Patel et al., 2005; Fitch and Rosenfeld, 2007; Pecenka et al., 2013). Considering this, the degraded accuracy for bimanual timing in the present study was likely caused by neural competition between meter processing and bimanual movements. Neural competition is caused by regional overlaps between two different brain functions (Colzato et al., 2021). According to that article, such competition enhances one function and simultaneously inhibits the other. Regarding the present study, both bimanual control and the processing of a metrical structure for complex auditory rhythms such as syncopation recruit the supplementary sensorimotor area (SMA, Nair et al. (2003) and Kasdan et al. (2022)). Because the similarity in brain activities between individual tasks can predict the extent to which performance will decline during multitasking (Nijboer et al., 2014), meter and pulse perception and bimanual movements might compete for identical neural resources in the SMA. Although we did not monitor actual brain activity in the present study, such neural competition likely degraded the meter and pulse perception for syncopation, leading to more inaccurate SMS in the BM condition than the ND condition.

In spite of syncopation and the absence of tactile and visual feedback, the index ASY in the present study, the temporal relationship between the timings of stimuli and movements, did not differ considerably from that typically reported in previous studies using table tapping to isochronous metronomes [e.g., Aschersleben et al. (2001)], i.e., we found common phenomenon referred to as “negative mean asynchrony (Repp, 2005).” In contrast, some studies using finger flexion in the air reported different tendencies of asynchrony, e.g., the maximal flexion occurred after beat onsets in Miura et al. (2016). Such a difference might be caused by the instructions and training in the present experiments. In the first step of the practice session, participants were engaged in table tapping. In the following step, we asked participants to flex their fingers in the air exactly in the same manner as in the preceding step. In other words, participants were trained to replicate table tapping in the air. The present study highlighted the importance of the instructions and training provided to participants, which could alter the tactics of SMS and the results.

This study has several limitations. First, some parameters of the auditory stimulus and the experimental procedure might have affected the results. In fact, the frequency of movements, the degree of syncopation, and the sensory modality for rhythm perception are known to affect the quality of SMS (Patel et al., 2005; Bravi et al., 2017; Witek et al., 2017; Zalta et al., 2020). Compared to similar previous studies, the present experiments were unusual in using syncopation and eliminating tactile and visual feedback, which might have caused unique results. In other words, when providing isochronous metronomes or such sensory feedback, the parallel execution of SMS in the head or the non-dominant hand might affect SMS in the dominant hand differently. As well, the effects of head SMS on finger SMS might change when the frequency of movements, i.e., the tempo of auditory stimuli, exceeds a second because of the difference in the neural mechanisms between suprasecond and subsecond timing (Meck, 2005; Hayashi et al., 2014). Second, participant demographic information, such as the years of music training, age of musical training onset, and types of musical instruments played might influence SMS performance (Bailey and Penhune, 2010; Krause et al., 2010; Repp, 2010). Third, the temporal relationship between sensory inputs and motor execution may alter SMS performance. Some studies have demonstrated that antiphase bimanual movements to sensory rhythms show a different quality of synchronization compared with inphase bimanual movements (Keller and Repp, 2004; Blais et al., 2015). The present study did not focus on these factors because we examined the positive effects of head movements on normal musical activities in the general population. Future studies should address the potential of these factors to interact with the present results. Fourth, the present experiments might have required additional attentional demand to suppress spontaneous head movements in the WN and BM conditions, which could have modulated the SMS performance. In these conditions, however, participants wore a U-shaped cushion around their neck to reduce the voluntary effort for fixing their heads as much as possible. Hence, we believe that the influence of attentional demand suppressing head movements on SMS performance was slight or negligible. Finally, the results of the sensitivity analysis that included an outlier participant differed from that without him/her when comparing the ASY_abs_ between the ND and BM conditions in Exp. 2. According to visual inspection by the experimenter, the outlier participant had difficulty maintaining head SMS. Considering this, poor head SMS performance might have decreased the quality of finger SMS.

## 10 Conclusion

The present study examined the influence of head SMS to perceived pulses on the SMS of finger flexion executed in parallel, and revealed that head movements had a positive impact on the synchronization stability of finger flexion. Furthermore, we found that the parallel execution of head SMS and dominant hand finger SMS was more advantageous in terms of SMS accuracy compared with bimanual SMS. In addition, the correlational analyses indicated that head movements were implicated in finger flexion synchronization stability via a different mechanism from that of bimanual movements. These findings highlight the unique role of head movements in musical activities such as playing instruments or dancing. Furthermore, since some other species move their heads to music (Fitch, 2013), this study could support future research on the underlying mechanisms that produce such behavior in non-human animals.

## Data availability statement

The raw data supporting the conclusions of this article will be made available by the authors, without undue reservation.

## Ethics statement

The studies involving humans were approved by the Research Ethics Committee at Shonan Fujisawa Campus, Keio University. The studies were conducted in accordance with the local legislation and institutional requirements. The participants provided their written informed consent to participate in this study.

## Author contributions

RY: Conceptualization, Data curation, Formal analysis, Funding acquisition, Investigation, Methodology, Project administration, Resources, Software, Validation, Visualization, Writing – original draft. JU: Conceptualization, Funding acquisition, Methodology, Project administration, Supervision, Writing – review & editing.
