# Supplementary material for: Head movements induced by voluntary neck flexion stabilize sensorimotor synchronization of the finger to syncopated auditory rhythms
Source: Front Psychol. 2024 Jun 6;15:1335050. doi: 10.3389/fpsyg.2024.1335050 (PMC11188995; doi:10.3389/fpsyg.2024.1335050)
Supplement: Supplementary file 1 [file Data_Sheet_1.docx]

Supplementary Material

Head Movements Induced by Voluntary Neck Flexion Stabilize Sensorimotor Synchronization of the Finger to Syncopated Auditory Rhythms

Ryoichiro Yamazaki *, Junichi Ushiyama *

*** Correspondence:**

Junichi Ushiyama: ushiyama@sfc.keio.ac.jp

Ryoichiro Yamazaki: zackysdc@sfc.keio.ac.jp

# Supplementary Data

Supplementary Material should be uploaded separately on submission. Please include any supplementary data, figures and/or tables.

Supplementary material is not typeset so please ensure that all information is clearly presented, the appropriate caption is included in the file and not in the manuscript, and that the style conforms to the rest of the article.

# Supplementary Figures and Tables

For more information on Supplementary Material and for details on the different file types accepted, please see [here](https://www.frontiersin.org/guidelines/author-guidelines#supplementary-material).

## Supplementary Figures

**
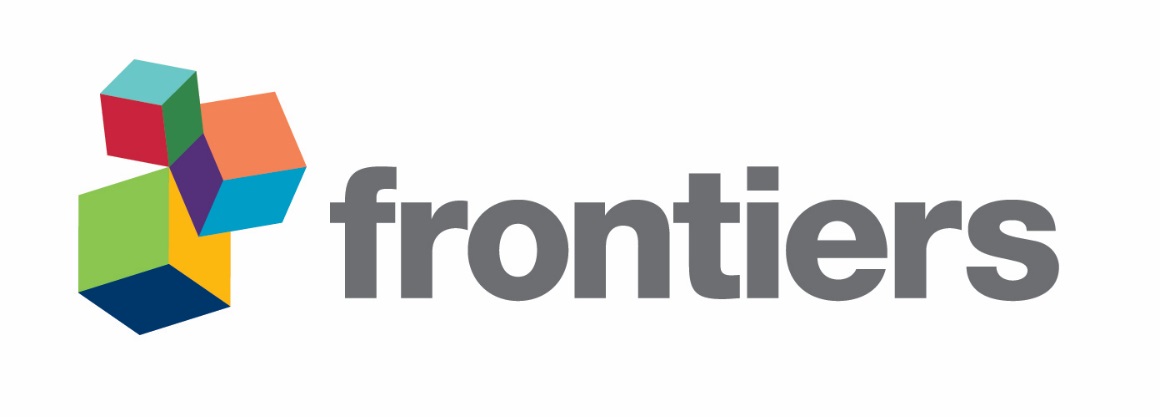
**

**Supplementary Figure 1.** The figure legends are required to have the same font as the main text, 12 point normal Times New Roman, single spaced. Please use a single paragraph for each legend and prepare the figures keeping in mind the PDF layout.

**TABLE S1 |** Descriptive statistics for Exp. 1. WN, Without Nodding condition; ND, Nodding condition; SEM, standard error of means; ASY, asynchrony of synchronization; ASY_SD_, standard deviation of ASY; ASY_abs_, absolute values of ASY.

|  |  | **WN** |  | **ND** |  |
| --- | --- | --- | --- | --- | --- |
|  |  |  |  |  |  |
| ASY (SEM), ms |  | -32.46 |  | -22.67 |  |
|  |  | (-37.98) |  | (45.16) |  |
|  |  |  |  |  |  |
| ASY_SD_ (SEM), ms |  | 32.58 |  | 30.09 |  |
|  |  | (7.79) |  | (5.58) |  |
|  |  |  |  |  |  |
| ASY_abs_ (SEM), ms |  | 49.85 |  | 48.45 |  |
|  |  | (20.34) |  | (23.51) |  |

**TABLE S2 |** Correlation between ASY_SD_ and the PLI in the ND condition in Exp. 1. PLI, phase locking index; ND, Nodding condition; CI, confidence interval; *p*_FDR_, p value after FDR correction; ASY_SD_, standard deviation of asynchrony of synchronization.

| **PLI** |  | ***r*** |  | **95% CI of *r*** | |  | ***p*_FDR_** |
| --- | --- | --- | --- | --- | --- | --- | --- |
| PLI_Head-Pulse_ |  | -0.85 |  | -0.94 | -0.66 |  | < 0.001 |
| PLI_Head-Dom_ |  | -0.11 |  | -0.53 | 0.35 |  | 0.64 |

**TABLE S3 |** Correlation between ASY_SD_ and the PLI with outliers in the ND condition in Exp. 1. PLI, phase locking index; CI, confidence interval; ND, Nodding condition; *p*_FDR_, p value after FDR correction; ASY_SD_, standard deviation of asynchrony of synchronization.

| **PLI** |  | ***r*** |  | **95% CI of *r*** | |  | ***p*_FDR_** |
| --- | --- | --- | --- | --- | --- | --- | --- |
| PLI_Head-Pulse_ |  | -0.86 |  | -0.93 | -0.70 |  | < 0.001 |
| PLI_Head-Dom_ |  | -0.11 |  | -0.49 | 0.30 |  | 0.59 |

**TABLE S4 |** Descriptive statistics in Exp. 2. BM, Bimanual condition; ND, Nodding condition; SEM, standard error of means; ASY, asynchrony of synchronization; ASY_SD_, standard deviation of ASY; ASY_abs_, absolute values of ASY.

|  |  | BM |  | ND |  |
| --- | --- | --- | --- | --- | --- |
|  |  |  |  |  |  |
| ASY (SEM), ms |  | -36.75 |  | -24.74 |  |
|  |  | (46.35) |  | (30.13) |  |
|  |  |  |  |  |  |
| ASY_SD_ (SEM), ms |  | 31.16 |  | 31.77 |  |
|  |  | (8.28) |  | (8.22) |  |
|  |  |  |  |  |  |
| ASY_abs_ (SEM), ms |  | 53.43 |  | 42.50 |  |
|  |  | (30.72) |  | (15.32) |  |

**TABLE S5 |** Descriptive statistics with outliers in Exp. 2. BM, Bimanual condition; ND, Nodding condition; SEM, standard error of means; ASY, asynchrony of synchronization; ASY_SD_, standard deviation of ASY; ASY_abs_, absolute values of ASY.

|  |  | BM |  | ND |  |
| --- | --- | --- | --- | --- | --- |
|  |  |  |  |  |  |
| ASY (SEM), ms |  | -39.42 |  | -28.43 |  |
|  |  | (47.41) |  | (35.01) |  |
|  |  |  |  |  |  |
| ASY_SD_ (SEM), ms |  | 31.74 |  | 32.32 |  |
|  |  | (8.62) |  | (8.52) |  |
|  |  |  |  |  |  |
| ASY_abs_ (SEM), ms |  | 55.50 |  | 45.55 |  |
|  |  | (31.88) |  | (21.60) |  |

**Table S6 |** Correlation between behavioral measurements and the PLI in Exp. 2. ASY, asynchrony of synchronization; ASY_SD_, standard deviation of ASY; ASY_abs_, absolute values of ASY; PLI, phase locking index; CI, confidence interval; *p*_FDR_, p value after FDR correction.

|  |  | ***r*** |  | **95% CI of *r*** | |  | ***p*_FDR_** |
| --- | --- | --- | --- | --- | --- | --- | --- |
| **ASY_SD_** |  |  |  |  |  |  |  |
| PLI_Head-Pulse_ |  | -0.85 |  | -0.94 | -0.65 |  | < 0.001 |
| PLI_Head-Dom_ |  | -0.44 |  | -0.74 | 0.00 |  | 0.15 |
| PLI_Non-Pulse_ |  | -0.25 |  | -0.62 | 0.22 |  | 0.86 |
| PLI_Non-Dom_ |  | 0.19 |  | -0.28 | 0.58 |  | 0.64 |
| **ASY_abs_** |  |  |  |  |  |  |  |
| PLI_Head-Pulse_ |  | -0.21 |  | -0.59 | 0.26 |  | 0.46 |
| PLI_Head-Dom_ |  | -0.29 |  | -0.65 | 0.17 |  | 0.31 |
| PLI_Non-Pulse_ |  | -0.25 |  | -0.62 | 0.22 |  | 0.58 |
| PLI_Non-Dom_ |  | 0.14 |  | -0.32 | 0.55 |  | 0.67 |

**Table S7 |** Correlation between behavioral measurements and the PLI with outliers in Exp. 2. ASY, asynchrony of synchronization; ASY_SD_, standard deviation of ASY; ASY_abs_, absolute values of ASY; PLI, phase locking index; CI, confidence interval; *p*_FDR_, p value after FDR correction.

|  |  | ***r*** |  | **95% CI of *r*** | |  | ***p*_FDR_** |
| --- | --- | --- | --- | --- | --- | --- | --- |
| **ASY_SD_** |  |  |  |  |  |  |  |
| PLI_Head-Pulse_ |  | -0.88 |  | -0.94 | -0.74 |  | < 0.001 |
| PLI_Head-Dom_ |  | -0.30 |  | -0.62 | 0.10 |  | 0.16 |
| PLI_Non-Pulse_ |  | -0.23 |  | -0.56 | 0.18 |  | > 0.27 |
| PLI_Non-Dom_ |  | 0.04 |  | -0.35 | 0.42 |  | > 0.85 |
| **ASY_abs_** |  |  |  |  |  |  |  |
| PLI_Head-Pulse_ |  | -0.36 |  | -0.66 | 0.03 |  | 0.11 |
| PLI_Head-Dom_ |  | -0.40 |  | -0.68 | -0.02 |  | 0.08 |
| PLI_Non-Pulse_ |  | -0.17 |  | -0.52 | 0.24 |  | > 0.41 |
| PLI_Non-Dom_ |  | -0.04 |  | -0.42 | 0.35 |  | > 0.85 |
